# FGF2 Functions in H_2_S’s Attenuating Effect on Brain Injury Induced by Deep Hypothermic Circulatory Arrest in Rats

**DOI:** 10.1007/s12033-023-00952-3

**Published:** 2023-11-02

**Authors:** Yu-Xiang Zhu, Qin Yang, You-Peng Zhang, Zhi-Gang Liu

**Affiliations:** 1 Department of Cardiovascular Surgery, TEDA International Cardiovascular Hospital, Chinese Academy of Medical Sciences and Peking Union Medical College, 61 No. 3 Ave, Binhai District, Tianjin, 300457 People’s Republic of China; 2 Center for Basic Medical Research, TEDA International Cardiovascular Hospital, Chinese Academy of Medical Sciences and Peking Union Medical College, Binhai District, Tianjin, 300457 People’s Republic of China

**Keywords:** H_2_S, DHCA, FGF2, Brain Injury

## Abstract

Deep hypothermic circulatory arrest (DHCA) can protect the brain during cardiac and aortic surgery by cooling the body, but meanwhile, temporary or permanent brain injury may arise. H_2_S protects neurons and the central nervous system, especially from secondary neuronal injury. We aim to unveil part of the mechanism of H_2_S’s attenuating effect on brain injury induced by DHCA by exploring crucial target genes, and further promote the clinical application of H_2_S in DHCA. Nine SD rats were utilized to provide histological and microarray samples, and further the differential expression analysis. Then we conducted GO and KEGG pathway enrichment analyses on candidate genes. The protein-protein interaction (PPI) networks were performed by STRING and GeneMANIA. Crucial target genes’ expression was validated by qRT-PCR and western blot. Histological study proved DHCA’s damaging effect and H_2_S’s repairing effect on brain. Next, we got 477 candidate genes by analyzing differentially expressed genes. The candidate genes were enriched in 303 GO terms and 28 KEGG pathways. Then nine genes were selected as crucial target genes. The function prediction by GeneMANIA suggested their close relation to immunity. FGF2 was identified as the crucial gene. FGF2 plays a vital role in the pathway when H_2_S attenuates brain injury after DHCA. Our research provides more information for understanding the mechanism of H_2_S attenuating brain injury after DHCA. We infer the process might probably be closely associated with immunity.

**Supplementary Information:**

The online version contains supplementary material available at 10.1007/s12033-023-00952-3.

## Background

Deep hypothermic circulatory arrest (DHCA), also known as a stand-still technique which has been an integral part of cardiac and aortic surgery for decades [1], can protect the brain to some extent by cooling the body to temperatures between 14.1 and 20 °C to stop blood circulation and brain function for up to 40 min [2]. Meanwhile, it provides a bloodless operation site. However, a longer duration of circulatory arrest is associated with increased neurologic complications [3]. It is unpredictable whether the brain injuries are temporary or permanent after surgery with the aid of DHCA [4]. The injuries resulting from these conditions can manifest as obvious impairments like seizures [5], or more subtle ones such as compromised psychomotor function [6]. Prolonged durations of DHCA exceeding 20 min, especially surpassing 35 min, have a detrimental impact on the mid-term quality of life (QoL) of patients undergoing thoracic aorta surgery [7]. However, research about brain protection during DHCA is still limited.

H_2_S, or hydrogen sulfide, has gained recognition as a significant endogenous gaseous signaling molecule [8], which exists in blood and can be generated by two enzymes: cystathionine-γ-lyase & -β-synthetase [9]. It is widely acknowledged to have a crucial role in regulating signal transduction and various biological functions, including immunology [10] in human health and disorders by modifying proteins and small molecules via sulfhydration [11]. Functions of H_2_S encompass a wide range of roles, such as regulating functions in the cardiovascular system, exerting modulatory effects on various aspects of cellular metabolism, and playing regulatory roles in numerous health and disease conditions [12, 13]. H_2_S protects neurons and the central nervous system, especially from secondary neuronal injury. The endogenous level is significantly higher in the brain compared to peripheral tissues [14]. Exogenous H_2_S is also widely used in medical research, not only due to its putative therapeutic applications, but also because it is a tool to discover the potential physiological functions of endogenous H_2_S [15]. The administration of exogenous H_2_S has shown potential to enhance outcomes of cancer, tissue fibrosis diseases, diabetic-related diseases [16], and ischemia/reperfusion (I/R) injury by regulating autophagy [17]. It has also been proved that H_2_S could provide protection via interacting with oxytocinin in cases of physical and psychological trauma [18]. In circulatory shock, concentration of vascular sulfide might play a significant role [19]. As a neuroprotective agent, H_2_S donor NaHS induces a hibernation-like metabolic state and hypothermia [20]. Despite ongoing research, the precise protective mechanism of H_2_S in the context of DHCA remains insufficiently understood, and the association between H_2_S and DHCA is largely unexplored.

In this study, our objective was to investigate the essential target genes involved in H_2_S’s attenuating effect on brain injury resulting from DHCA. We aim to contribute insights into the pivotal molecular mechanisms underlying H_2_S-related neuroprotection. These insights might hold significant promise for advancing the clinical utilization of H_2_S in the context of DHCA.

## Materials and Methods

### Animals

Ethics Statement: The Institutional Ethics Review Board of TEDA International Cardiovascular Hospital provided approval for all animal experiments (ethic code: TICH-JY-20220715-2). Meticulous attention was dedicated to upholding the well-being, proper care, and ethical treatment of the animals, with the overarching goal of reducing any potential distress experienced by the animals during the study.

In this study, nine male Sprague-Dawley (SD) rats weighing 450–520 g were bought from Tianjin Aochen Laboratory Animal Sales Co., Ltd (Tianjin, China). The rats were randomly divided into three groups:

#### Control Group (n = 3)

In rats placed under general anesthesia, endotracheal intubation was conducted, and inhalation of 1.2% isoflurane anesthesia was administered. To maintain proper respiratory function, a ventilator was employed. Monitoring of blood pressure was established by inserting a 22G cannula into the left femoral artery, while a separate 22G cannula was introduced into the tail artery. The right jugular vein was cannulated with a purpose-designed multi-lumen catheter for drainage. The cardiopulmonary bypass (CPB) circuit comprised a dual roller pump (Stockert, München, Germany), a blood reservoir, a blood heat exchanger, and a blood oxygenator (Xi’an Xijing, Xi’an, China). The regulation of circulatory temperature was achieved using a Heater-Cooler System 3T (Stockert, München, Germany).

#### DHCA Group (n = 3)

Subsequent to the protocol executed in the control group, the core temperature of the rats was systematically reduced to 17 °C, thereby initiating a period of DHCA lasting 45 min under this specified core temperature. During the DHCA phase, the operation of the extracorporeal circuit was suspended, concomitant with the disconnection of the right jugular vein catheter and the blood reservoir from the circuitry. Cardiac arrest was induced through the controlled drainage of blood via the right jugular vein catheter, with the withdrawn blood being segregated. Following the restoration of extracorporeal circulation, the previously withdrawn blood was systematically reintroduced into the blood reservoir, accompanied by gradual and controlled rewarming. Following a period of 60 min of controlled rewarming, the core temperature of the rats was systematically restored to 35 °C, culminating in the cessation of extracorporeal circulation. Subsequently, the rats were subjected to a duration of observation lasting one hour, during which they remained under general anesthesia with mechanical ventilation support. Upon completion of the observation period, the subjects were humanely euthanized in accordance with established ethical procedures.

#### DHCA + H_2_S Group (n = 3)

Following the induction of brain injury through DHCA, the members received treatment involving the administration of the H_2_S donor NaHS (Rhawn, R014855, Shanghai, China). NaHS powder was dissolved in a solution of normal saline, yielding a concentration of 2 mg/ml. Consistent with the DHCA group’s established protocol, intraperitoneal injection of 8 mg/kg of NaHS was carefully administered, with this intervention taking place 30 min prior to the initiation of circulatory arrest.

### Histologic Study

After the rats were euthanized, all samples from DHCA group, DHCA + H_2_S group, and Control group were taken from the rats’ parietal cortex. To perform histological evaluation, we fixed and sectioned the tissues, and then stained them with hematoxylin-eosin (H&E).

### Microarray Analysis

The total RNA was extracted by TRIzol reagent (Invitrogen, 15,596,018, Carlsbad, CA, USA). The concentration of the RNA samples was determined by NanoDrop ND-1000 spectrophotometer (ThermoScientific, MA, USA). Then the protocol of microarray was the same as mentioned [21].

### Differential Gene Analysis

Each differential gene analysis was performed by “limma” package of R language (version 4.1.0). The criteria for screening differentially expressed genes (DEGs) were |log_2_FC| > 1 and p < 0.05. The figures were drawn by “ggplot2” and “pheatmap” packages.

### Functional Enrichment Analysis

We carried out Gene Ontology (GO) enrichment analysis and Kyoto Encyclopedia of Genes and Genomes (KEGG) pathway enrichment analysis on candidate genes. The analysis was done by “clusterProfiler” [22] package. The significantly enriched terms and pathways were screened by criteria of p.adjust < 0.05.

### Protein-Protein Interaction (PPI) Network Analysis

The protein-protein interaction (PPI) network of candidate genes was constructed by use of online database STRING (https://cn.string-db.org). Then we utilized Maximum Neighborhood Component (MNC) in cytoHubba plugin of Cytoscape (3.9.1) to select and visualize crucial target genes. After that, the target genes were input to GeneMANIA (https://genemania.org) to get their potential functions, interactions, pathways, co-expression, and co-localization.

### RNA Isolation and Real-Time Quantitative PCR (qRT-PCR)

To corroborate the findings derived from the analysis of DEGs, a qRT-PCR assay was conducted on a selection of five target genes. Total RNA was extracted utilizing the TRIzol reagent (Invitrogen, 15,596,018, Carlsbad, CA, USA), followed by the implementation of reverse transcription through the utilization of the HiScript III RT SuperMix for qPCR (+ gDNA wiper) kit (Nanjing Vazyme Biotech Co. Ltd, R323-01, Nanjing, China). The reaction mixture consisted of 2×Color SYBR Green qPCR Master Mix (Nanjing Vazyme Biotech Co. Ltd, Q712-03, Nanjing, China), with primer sequences as delineated in Table 1. The internal control gene employed was GAPDH (Glyceraldehyde-3-phosphate dehydrogenase). The qRT-PCR was executed using the LightCycler 480 system (F. Hoffmann-La Roche Ltd, Basel, Switzerland), employing a thermal profile encompassing initial denaturation at 95 °C for 3 min, followed by 40 cycles of denaturation at 95 °C for 10 s and annealing/extension at 60 °C for 30 s. Each sample underwent a triplicate examination. Data analysis was conducted utilizing 2^−ΔΔCT^ method.

**Table 1:** The nucleotide sequences of primers used in qRT-PCR

Target gene	Primer sequence
Fgf2-F	TGTCTCCCGCACCCTATCC
Fgf2-R	CACAACGACCAGCCTTCCAC
Edn1-F	GGGAACAGATGCCAGTG
Edn1-R	AAAGGAGGTCTTGATGCT
Cxcl11-F	CGGTTCCAGGCTTCGTTA
Cxcl11-R	GTCCAGGCACCTTTGTCC
Ntrk1-F	GGGGCTAACTCTGGTCAAT
Ntrk1-R	AGGGTGTAGTTCCCGTTGT
Ccl12-F	ATCTCCACCCTTCTTTG
Ccl12-R	AACTATCGCACTGTCCAT
GAPDH-F	GACATGCCGCCTGGAGAAAC
GAPDH-R	AGCCCAGGATGCCCTTTAGT

### Western Blot

Protein extraction was accomplished utilizing RIPA buffer (Beijing Solarbio Science & Technology Co., Ltd., R0020) supplemented with PMSF. Primary antibodies targeting FGF2 (DF6038, 1:1000) and β-actin (AC004, 1:10000) were procured from Affinity Biologicals Inc (Ancaster, ON, Canada) and ABclonal technology (Woburn, MA, USA) respectively. The secondary antibodies employed for FGF2 and β-actin, specifically HRP-Goat Anti-Rabbit IgG H&L (ab97051, 1:10000) and HRP-Goat Anti-Mouse IgG H&L (AS003, 1:10000), were sourced from Abcam (Cambridge, UK) and ABclonal correspondingly. Subsequently, the procedural steps were aligned with those elucidated in the referenced article [19]. To quantify the relative band intensity, ImageJ software was employed.

## Results

### Histological Evidence of H_2_S Attenuating Brain Injury Caused by DHCA

**Fig. 1:**
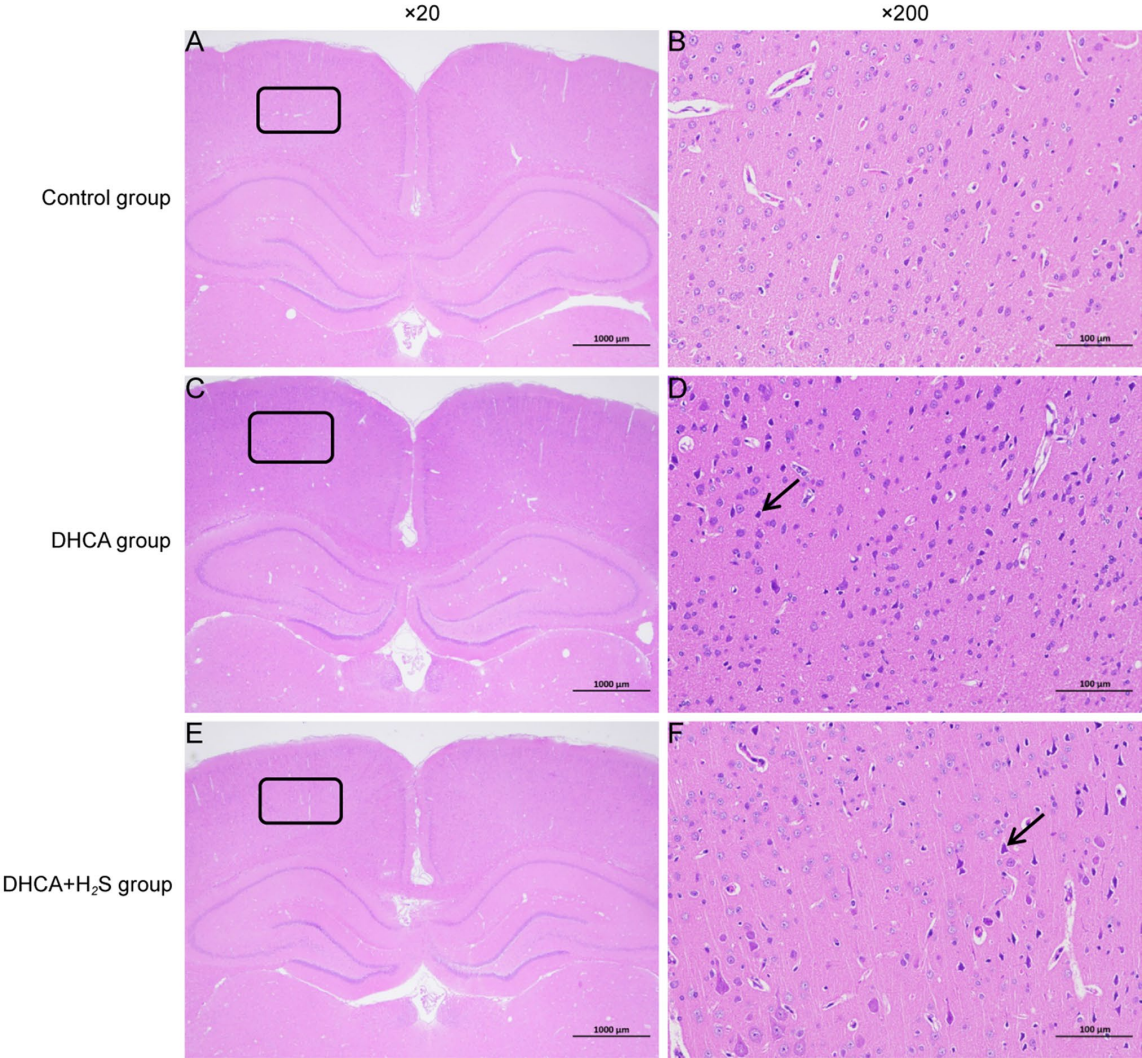
Hematoxylin-eosin staining of the brain sections. **A**, **B** Control group × 20 and × 200. In the parietal cortex tissue, cortical neurons are in regular shape, densely arranged, with large and round nuclei, less chromatin, and obvious nucleoli. No obvious abnormalities were found. **C**, **D** DHCA group × 20 and × 200. In the parietal cortex tissue, a large number of neurons in the cortex are pyknotic and deeply stained, the boundary between the nucleus and cytoplasm is unclear, and the basophilia is enhanced (black arrow). **E**, **F** DHCA + H_2_S group × 20 and × 200. In the parietal cortex tissue, a small amount of neurons in the cortex can be seen to be pyknotic and deeply stained, with unclear nucleocytoplasmic boundaries and enhanced basophilicity (black arrow)

To substantiate the occurrence of brain injury resulting from DHCA and the attenuating effect exerted by H_2_S, histological analysis involving hematoxylin-eosin staining of the parietal cortex was performed. In the control group, neurons appeared structurally intact, featuring discernible nucleoli within cytoplasm-rich environments (Fig. 1A and B). Conversely, the DHCA group exhibited irregularly contoured and pyknotic neurons (Fig. 1C and D). Notably, in the DHCA + H_2_S group, a noticeable reduction in the proportion of aberrant neurons was evident (Fig. 1E, F). This collective evidence proved the corroborative impact of DHCA and H_2_S on brain tissue damage and subsequent reparative processes.

### Identification of 477 Potential Candidate Genes of H_2_S’s Effect on Brain Injury Induced by DHCA

**Fig. 2:**
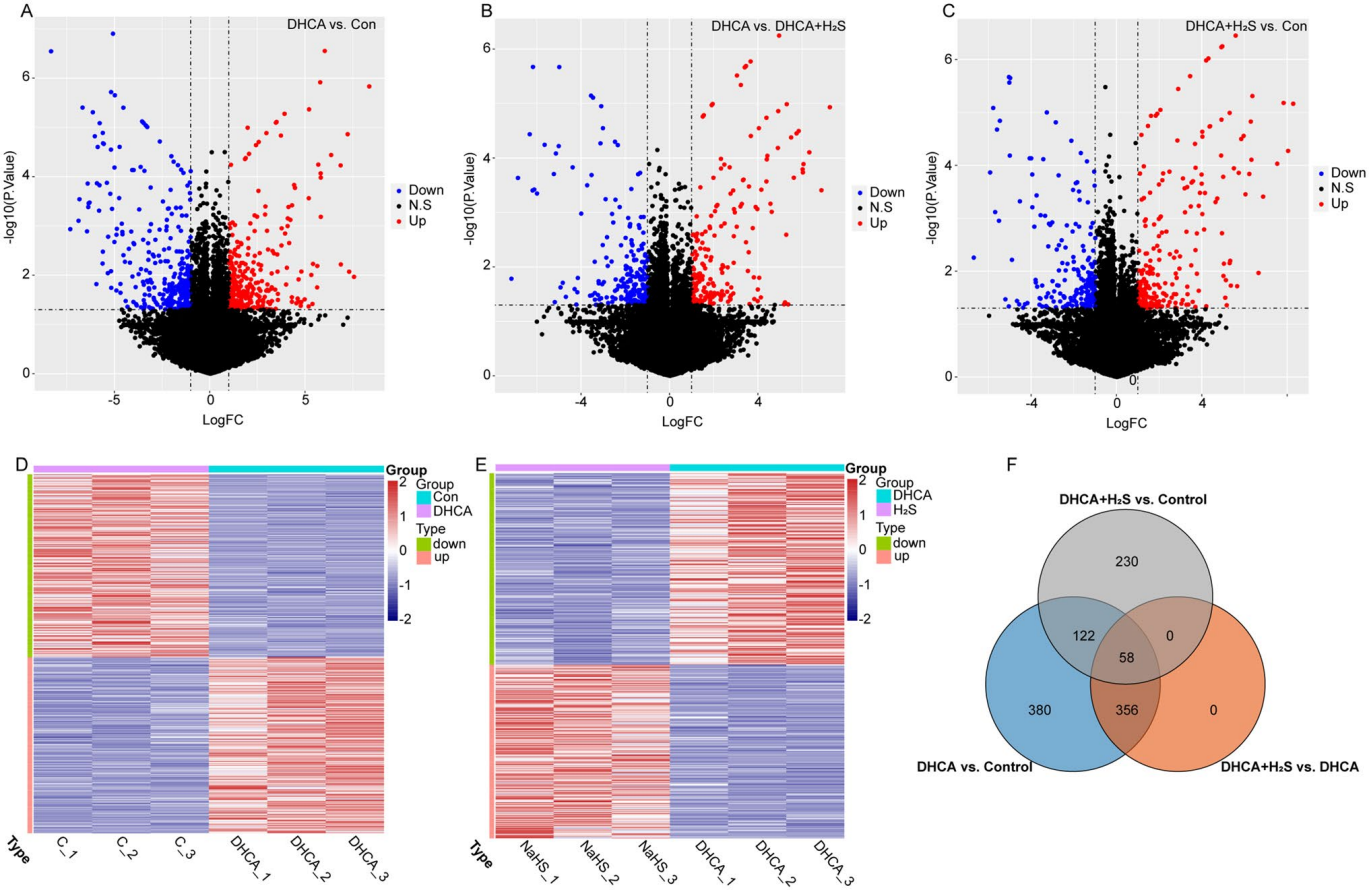
Differential gene expression analysis. **A** Fold change plotted against adjusted p-value (|log_2_FC| > 1 and p < 0.05). DEGs between DHCA group and control group. **B** DEGs between DHCA + H_2_S group and DHCA group. **C** DEGs between DHCA + H_2_S group and control group. **D** Heatmap of gene expression of DHCA group and control group. Red ones mean genes are relatively higher expressed and blue ones relatively lower. **E** Heatmap of gene expression of DHCA + H_2_S group and DHCA group. **F** DEGs among different groups

To investigate the mechanisms underlying the attenuating effect of H_2_S on brain injury induced by DHCA, we conducted a differential gene expression analysis on the three groups of samples, yielding results summarized in Table 2. Specifically, a total of 622 DEGs were identified when comparing the DHCA group to the control group. Among these DEGs, 305 were upregulated, while 317 were downregulated (Fig. 2A). Additionally, focusing on the interplay between H_2_S treatment and DHCA, we identified a set of 414 DEGs when contrasting the DHCA group to the DHCA + H_2_S group. 197 of the 414 DEGs exhibited upregulation, while 217 displayed downregulation (Fig. 2B). Furthermore, discerning the impact of H_2_S treatment, we identified 410 DEGs between the DHCA + H_2_S group and the control group, with 211 genes upregulated and 199 downregulated (Fig. 2C). These expression levels of DEGs among different groups were statistically significant (Fig. 2D and E).

**Table 2:** Differential gene expression analysis

	Upregulated	Downregulated
DHCA vs. Control	305	317
DHCA + H_2_S vs. DHCA	197	217
DHCA + H_2_S vs. Control	211	199

We aimed to find genes underpinning H_2_S’s attenuating effect on brain injury induced by DHCA, so we made a crossover analysis on DEGs between DHCA vs. Control group and DHCA vs. DHCA + H_2_S group. Then the DEGs between DHCA + H_2_S vs. Control group were excluded from the overlapping genes. Finally, we got 477 potential candidate genes (Fig. 2F, Table S1).

### Potential Functions and Pathways of Candidate Genes About H_2_S’s Effect on Attenuating Brain Injury

**Fig. 3:**
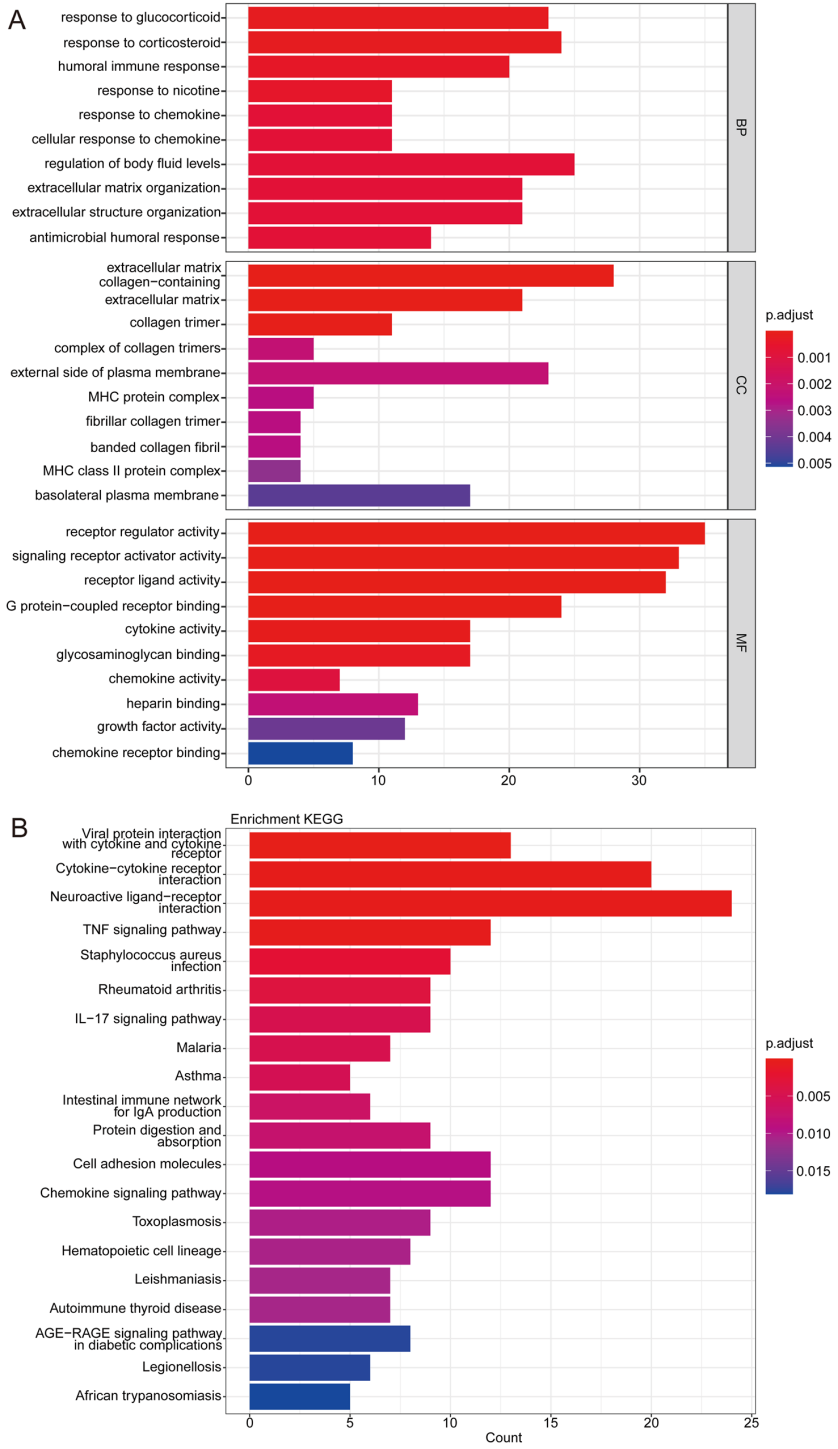
Gene function enrichment analysis. **A** Gene Ontology enrichment analysis. Three subfigures are 10 most significantly enriched GO terms in Biological Process (BP), Cellular Component (CC) and Molecular Function (MF) respectively. **B** KEGG pathway enrichment analysis. Top 20 significantly enriched pathways are shown here

To discover potential functions of the candidate genes, GO and KEGG pathway enrichment analyses were done on the 477 candidate genes relating to H_2_S’s role in attenuating brain injury induced by DHCA. Our results indicated that the 477 candidate genes were significantly enriched in 303 GO terms, and the top 10 significantly enriched GO terms of three aspects were shown in Fig. 3A respectively, containing response to glucocorticoid, response to corticosteroid, regulation of body fluid levels, extracellular matrix, receptor regulator activity, signaling receptor activator activity, etc. Additionally, totally 28 KEGG pathways were significantly enriched, and the top 20 significantly enriched KEGG pathways were shown in Fig. 3B. The entire results were attached in Table S2 and Table S3.

### Protein-Protein Interaction (PPI) Network Analysis of H_2_S’s Brain Protection Associated Genes

**Fig. 4:**
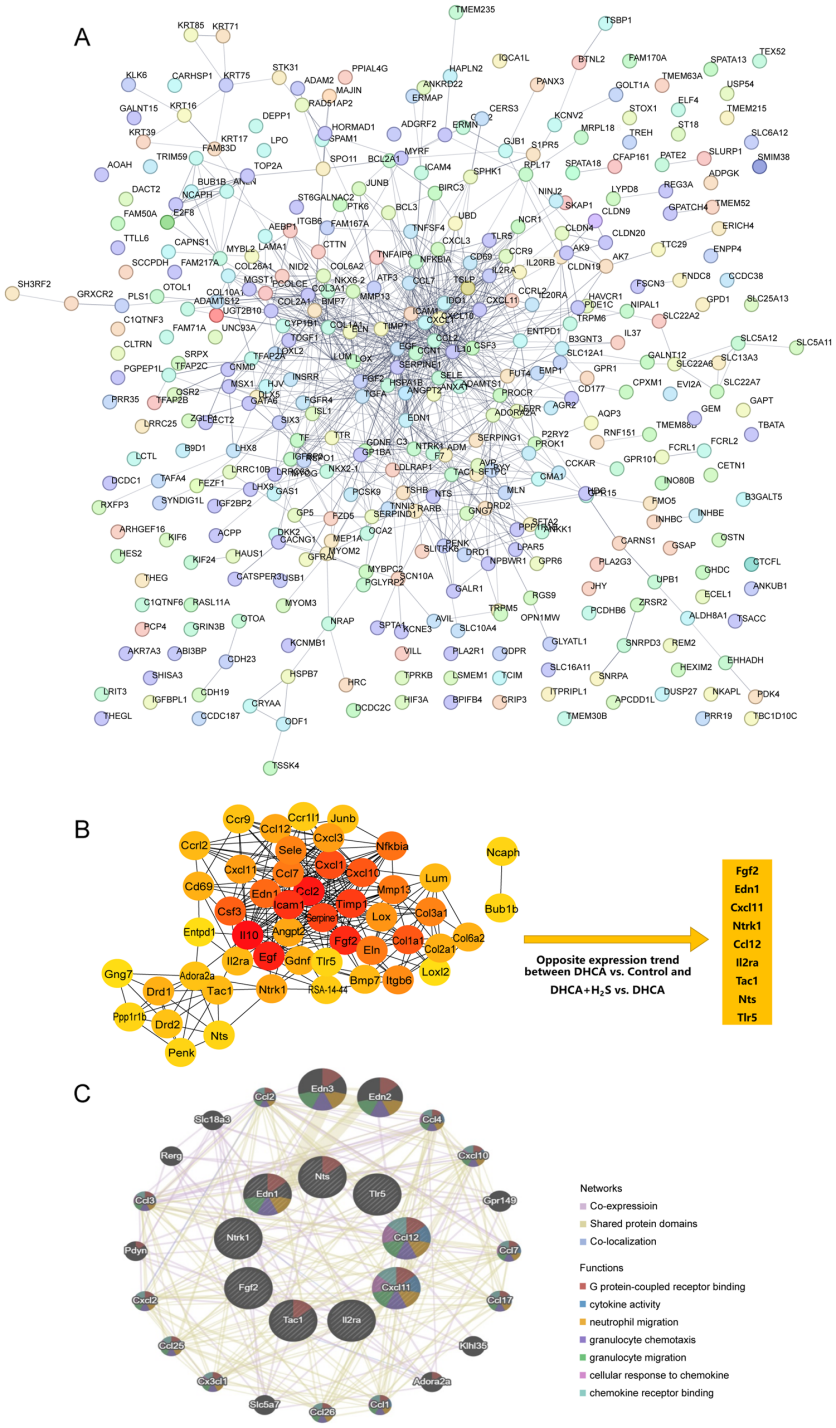
PPI network analysis. **A** The PPI network of 477 candidate genes. **B** The PPI network of top 50 genes. **C** 9 target genes’ network and functions predicted by GeneMANIA

For more potential related functions and pathways, the 477 candidate genes were input into the online database STRING for PPI network analysis. We constructed a network of 436 nodes and 820 edges, with PPI enrichment p-value < 1.0 × 10^−16^, and an average degree of 3.76 (Fig. 4A). The detailed data was attached in Table S4. Then top 50 genes were selected by MNC in cytoHubba plugin of Cytoscape (Fig. 4B, Table S5).

Next, we picked 9 genes whose expression trends were opposite between DHCA vs. Control group and DHCA + H_2_S vs. DHCA group (Table 3; Fig. 4B). They were FGF2 (Fibroblast growth factor 2), EDN1 (Endothelin 1), CXCL11 (C–X–C motif chemokine 11), NTRK1 (Neurotrophic receptor tyrosine kinase 1), CCL12 (Chemokine (C–C motif) ligand 12), IL2RA (Interleukin-2 receptor alpha chain/ CD25), TAC1 (Tachykinin-1), NTS (Neurotensin), and TLR5 (Toll-like receptor 5). Then we used GeneMANIA to develop these 9 genes’ potential co-expression, shared domains and co-localization. 20 closely related genes and potential functions were predicted (Fig. 4C). 7 functions with the least P value were displayed in the figure. They were granulocyte chemotaxis, G protein-coupled receptor binding, cytokine activity, neutrophil migration, granulocyte migration, cellular response to chemokine and chemokine receptor binding, suggesting their potential functions involving in a complex regulatory network in the process of H_2_S’ attenuating effect on brain injury caused by DHCA.

**Table 3:** Nine genes with opposite expression trends between DHCA vs. Control group and DHCA + H_2_S vs. DHCA group

Name	Score
Fgf2	32
Edn1	20
Cxcl11	14
Ntrk1	12
Ccl12	12
Il2ra	10
Tac1	10
Nts	7
Tlr5	7

### Expression Validation of Crucial Target Genes

Following an evaluation encompassing both differential expression analysis and expression trend analysis, nine genes emerged as focal points of interest. In order to corroborate the gene expression outcomes discerned from the microarray, a subsequent validation was undertaken through qRT-PCR targeting the five genes exhibiting the highest scores. FGF2 was focused to be the most crucial one not only because it scored the highest, but also because an abundance of literature provided evidence that FGF2 regulated neurogenesis after brain injury, for instance, virus amplicon vector carrying FGF-2 gene could recover the neuron proliferation and differentiation of FGF-2^−^ mice [23, 24].

**Fig. 5:**
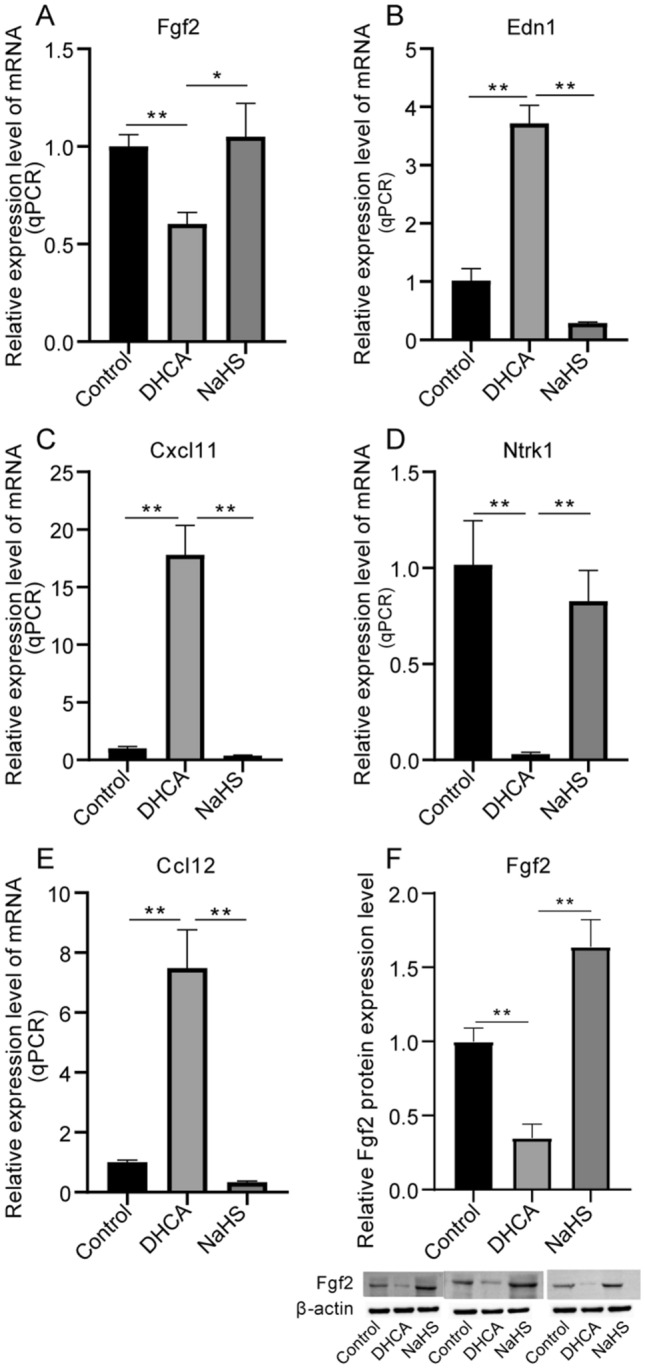
**A**–**E** The qRT-PCR results of FGF2, EDN1, CXCL11, NTRK1 and CCL12. **F** Western blot result of FGF2

Remarkably, the gene expression trends shown from the qRT-PCR outcomes and the transcriptome data exhibited harmonious alignment. Specifically, the expression levels of FGF2 and NTRK1 were notably lower in the DHCA group compared to the DHCA + H_2_S group (Fig. 5A and D). In contrast, EDN1, CXCL11, and CCL12 demonstrated significantly elevated expression in the DHCA group relative to the DHCA + H_2_S group (Fig. 5B, C and E). Of equal significance, the Western blot results pertaining to FGF2 mirrored the patterns evident in both qRT-PCR and microarray analyses (Fig. 5F). Collectively, the experimental findings provided further validation for the pivotal role of the central hub gene, FGF2, in mediating the effect of H_2_S in attenuating brain injury caused by DHCA.

## Discussion

In this study, firstly via histological evidence, we demonstrated DHCA could cause brain injury and H_2_S could attenuate the injury. Then 477 candidate genes related to H_2_S’s attenuating effect on brain injury induced by DHCA were screened from three groups of DEGs, followed by function enrichment analysis and PPI network analysis. Finally, 9 genes were selected as target genes, and FGF2 was the most crucial one.

To gain insight into the mechanism of H_2_S’s attenuating effect on brain injury induced by DHCA, we conducted function enrichment analysis. GO enrichment analysis of 477 candidate genes indicated that the top 10 enriched terms were focused on immune response, response to glucocorticoid and corticosteroid, collagen, chemokine, receptor, etc. Meanwhile, KEGG pathway enrichment analysis enriched top20 pathways such as cytokine − cytokine receptor interaction and TNF signaling pathway. H_2_S fulfills numerous functions in immunity including innate immunity and adaptive immunity [10]. For example, it can protect against tumor necrosis factor-α (TNF-α) induced neuronal cell apoptosis [25], and prevent the increase in monocyte adhesion induced by TNF-α [26]. H_2_S is involved in macrophage activation and inflammasome formation, which contributes to macrophage chemotaxis, polarization, adhesion and apoptosis [27]. The relationship between H_2_S and chemokine/chemokine receptors has been attracting more attention in recent years [28]. Chemokines may serve as mediators for the pro-inflammatory effects of H_2_S [29]. The same as H_2_S, low temperature also protects the brain from injury by immune-inflammatory processes [30]. We reasonably infer that one of the mechanisms of H_2_S’s protecting brain during DHCA is regulating immunity. As a synthetic glucocorticoid, high-dose methylprednisolone can be used preoperatively to attenuate the cerebral response to DHCA [31]. And H_2_S is closely related to glucocorticoid by negatively regulating glucocorticoid or glucocorticoid receptor α [32]. It is not hard to understand why collagen-related pathways are enriched. H_2_S plays a role in modulating vascular tone. It has complex effects on vascular walls in the systemic circulation and cerebrovascular region [33]. The endogenous pathway of H_2_S is implicated in the regulation of excessive vascular collagen in Spontaneously Hypertensive (SHR) rats [34].

Aiming to focus the research on a more accurate scope, we identified 9 target genes, FGF2, EDN1, CXCL11, NTRK1, CCL12, IL2RA, TAC1, NTS, and TLR5 whose expression trends were opposite between DHCA vs. Control group and DHCA + H_2_S vs. DHCA group. The gene with the highest score, FGF2 is a member of the fibroblast growth factor family [35]. As its name tells, it is essential in processes about growing, such as cell division, cell differentiation, cell survival and cell migration [36, 37] and functions as a potent mitogen in vitro [38]. FGF2 plays a vital role in varieties of biological processes, such as morphogenesis, embryonic development, tissue repair, tumor growth and invasion, and functions in varieties of diseases, e.g., chronic kidney disease (CKD), cancers [39], osteoporosis [40], and traumatic brain injury [41]. In addition, as a crucial immunity regulator in the tumor micro environment, FGF2 affects macrophage programming and can alter macrophage polarization, tumor immunity and growth [42]. The application of a wound healing system is recognized as a significant factor in promoting wound regeneration. This system operates by releasing H_2_S, which in turn induces the expression of the M2 macrophage phenotype, thereby contributing to the healing process [43]. The relationship between H_2_S and FGF2 likely exhibits proximity during the wound healing process. As H_2_S operates within physiological and pathological mechanisms, FGF2 frequently assumes a role along the pathway [44, 45]. In the context of mitigating brain injury induced by DHCA, it can be inferred that the beneficial effects of H_2_S are accompanied by a significant contribution from FGF2. What is more, In a study investigating the molecular and pharmacological mechanisms of N-ethylmaleimide (NEM) in inhibiting peripheral nerve degeneration (PND) by targeting Schwann cells through a H_2_S-pathway-dependent mechanism, EDN1 is discovered to be related to oxidative stress [46]. And CCL12 has been found to be one of the chemokines functioning in the mechanism of skeletal muscle regeneration after H_2_S therapy [47]. These genes are valuable for next step research on H_2_S’s attenuating effect on brain injury after DHCA.

After the 9 target genes were selected, we wanted to interpret how they serve in the pathways, so it was necessary to obtain their potential functions. From the GeneMANIA report, some of the 9 target gene’s functions were predicted. The most significant 7 ones were shown in Fig. 4C. They were G protein-coupled receptor binding, cytokine activity, neutrophil migration, granulocyte chemotaxis, granulocyte migration, cellular response to chemokine, and chemokine receptor binding. In an in vitro study investigating the effects of H_2_S, it has been demonstrated that HS- promotes the short-term survival of neutrophils by inhibiting caspase-3 cleavage and p38 MAP kinase phosphorylation. Furthermore, it facilitates inflammatory processes’ resolution and prevents the onset of new ones [48]. However, H_2_S is not only anti-inflammatory, it is also pro-inflammatory in different animal models and cell culture systems [49]. An example highlighting the significant pro-inflammatory role of H_2_S involves its regulation of the severity of pancreatitis and its association with lung injury [50]. Anyway, these studies are consistent with the functions predicted by GeneMANIA revealing a close relationship between these 9 genes and immunology.

The findings of this study hold promise for improving our understanding of the mechanisms underlying the attenuation of brain injury following DHCA by H_2_S. The identification of crucial target genes, with a particular emphasis on FGF2, sheds light on potential therapeutic targets for mitigating DHCA-induced brain injury. FGF2’s pivotal role in this pathway suggests that it may serve as a key molecular player in the protection of brain tissue during DHCA. The potential of H_2_S to control vascular tone, inflammation, oxidative stress, and apoptosis could prove highly beneficial in the therapeutic management of critical illness [51]. Our study opens up possibilities for developing novel treatments or interventions that harness the neuroprotective properties of H_2_S and its downstream regulatory genes. Further research into modulating FGF2 and related targets may offer valuable strategies to enhance patient outcomes in clinical settings where DHCA is utilized, such as cardiac surgeries requiring cardiopulmonary bypass. However, our research utilized an animal model, and while it provides valuable insights, findings may not fully extrapolate to the complexity of human DHCA scenarios. Moreover, further investigations are needed to assess the long-term effects and clinical implications of H_2_S treatment in the context of DHCA-induced brain injury. Ultimately, this study contributes to the broader goal of advancing clinical approaches to reduce brain injury associated with complex surgical procedures, potentially leading to improved patient well-being and quality of life.

## Conclusions

In this study, we got the transcriptomes of three groups of rats focusing on DHCA and H_2_S. A total of 477 candidate genes were screened, enriched on 303 GO terms and 28 KEGG pathways. Among the candidate genes, we selected 9 target genes whose expression trends were opposite between DHCA vs. Control group and DHCA + H_2_S vs. DHCA group. These genes might play vital roles in the pathway when H_2_S attenuates brain injury after DHCA. Among them, FGF2 deserved extra attention and we consider it as a crucial gene in the pathway of H_2_S attenuating brain injury induced by DHCA. The process might probably be closely associated with immunity. Our research provides more information for understanding the mechanism of H_2_S attenuating brain injury after DHCA. The findings could contribute to H_2_S’s clinical application in DHCA-related surgeries in the future as well.

## Supplementary Information

Below is the link to the electronic supplementary material.

Supplementary material 1 (XLSX 15.3 kb)

Supplementary material 2 (XLSX 40.5 kb)

Supplementary material 3 (XLSX 12.1 kb)

Supplementary material 4 (XLSX 115.6 kb)

Supplementary material 5 (XLSX 10.1 kb)

Supplementary material 6 (PDF 101 kb)

## Data Availability

The datasets analyzed are available in STRING database (https://cn.string-db.org). All data generated or analyzed during this study are included in this article.
