# Supplementary figures and images for: FGF2 Functions in H2S’s Attenuating Effect on Brain Injury Induced by Deep Hypothermic Circulatory Arrest in Rats
Source: Mol Biotechnol. 2023 Nov 2;66(12):3526–37. doi: 10.1007/s12033-023-00952-3 (PMC11564249; doi:10.1007/s12033-023-00952-3)

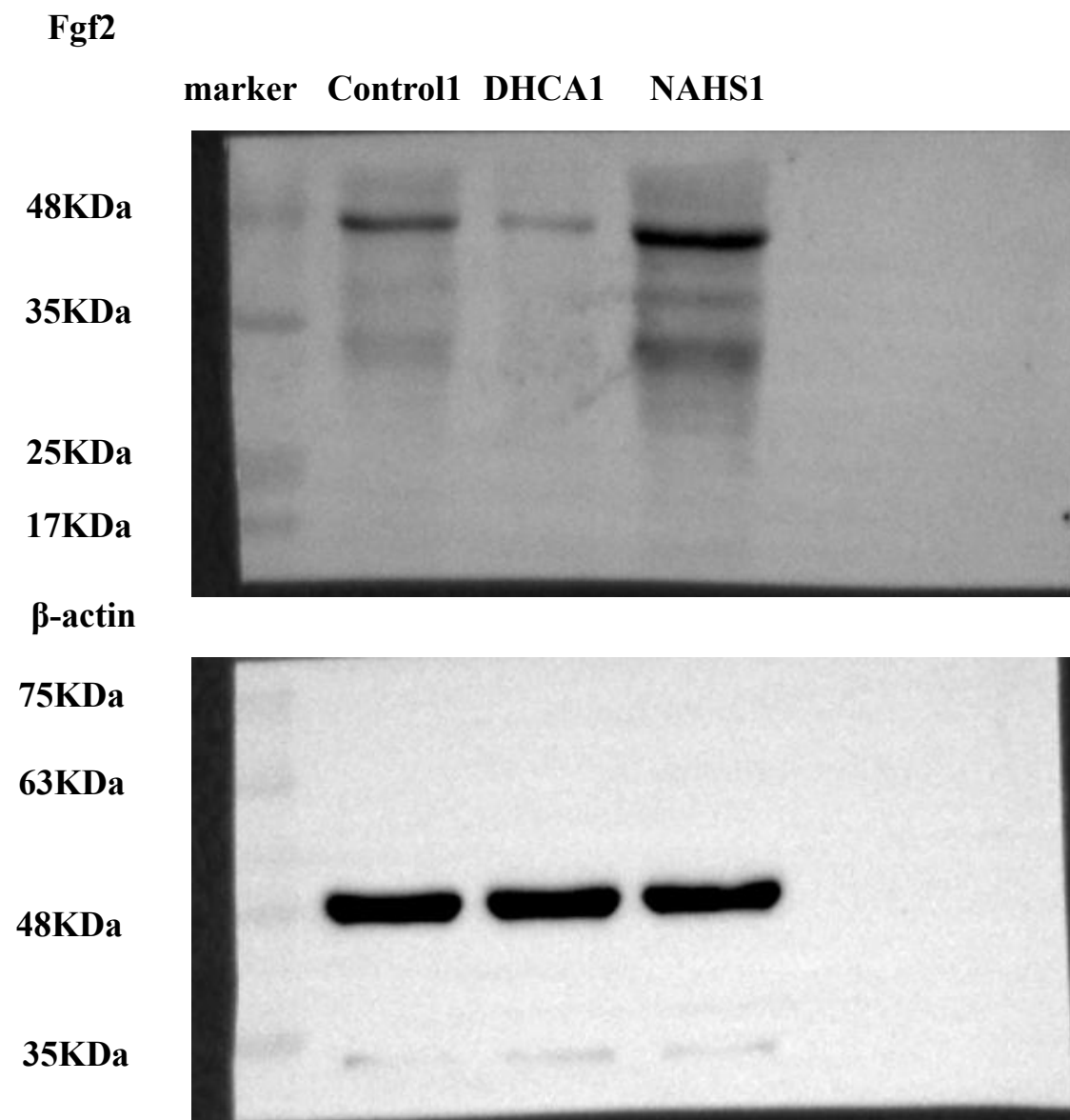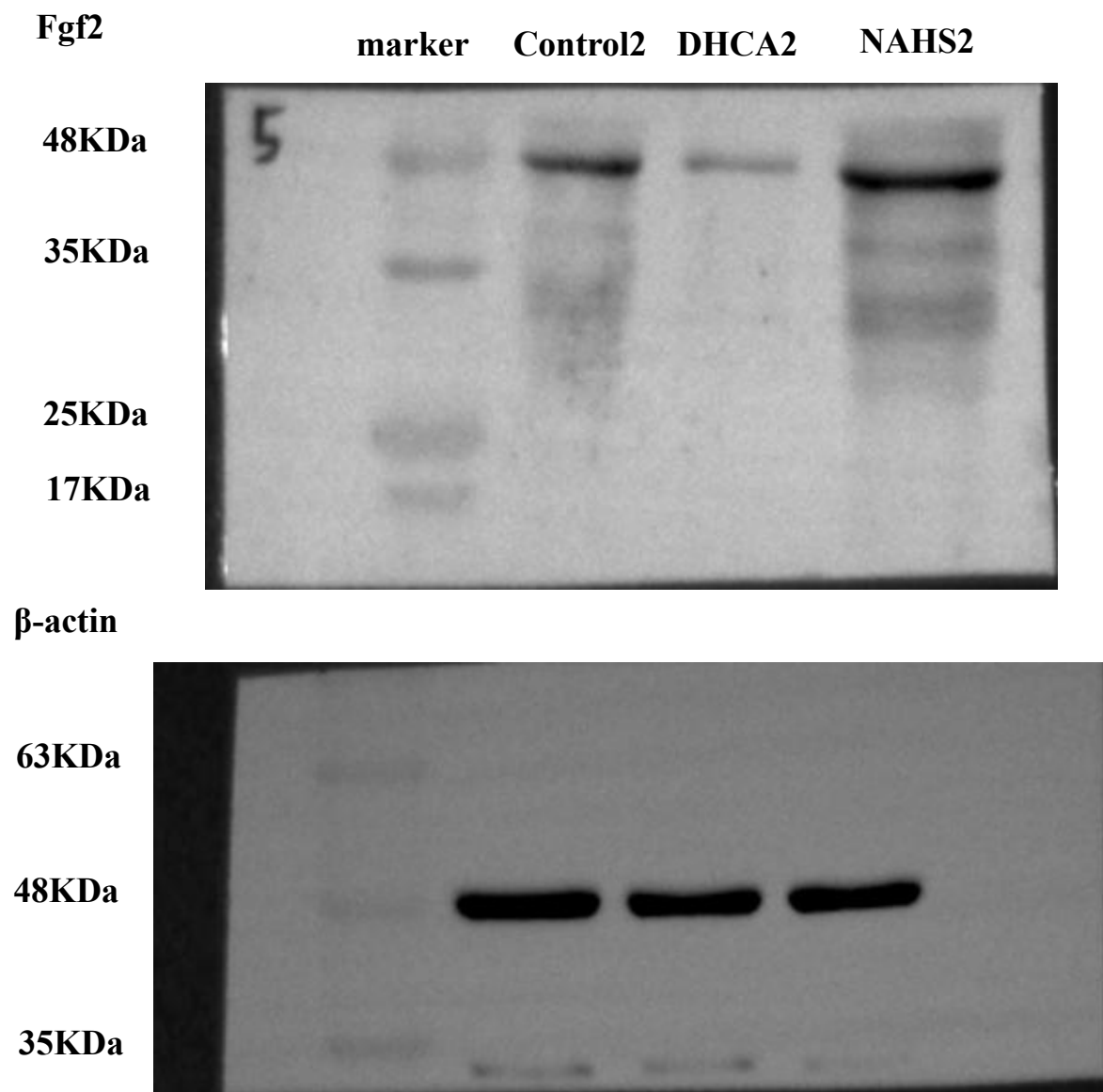

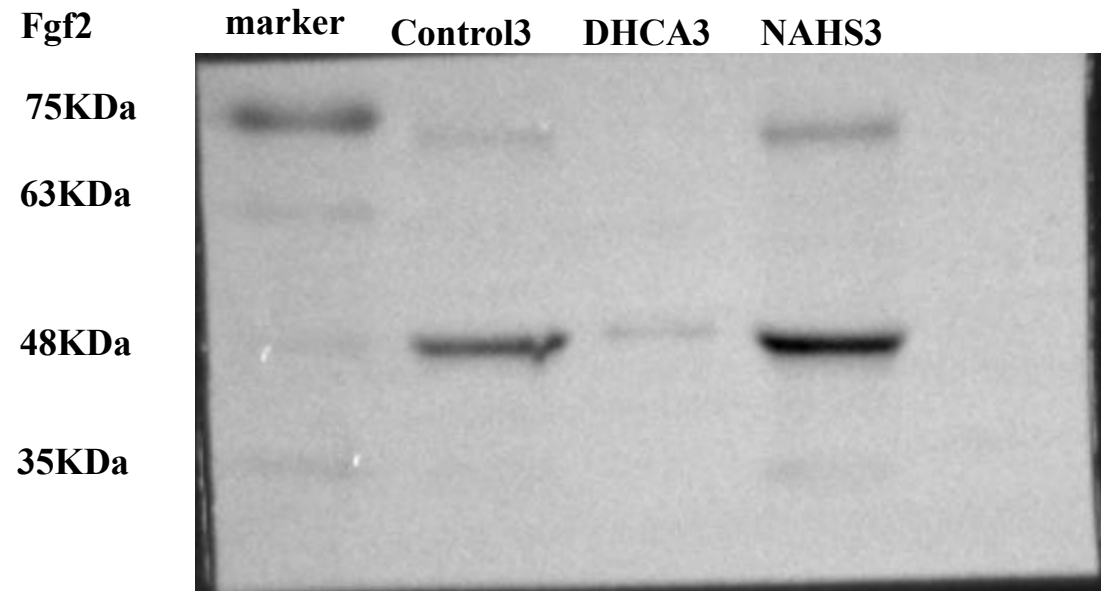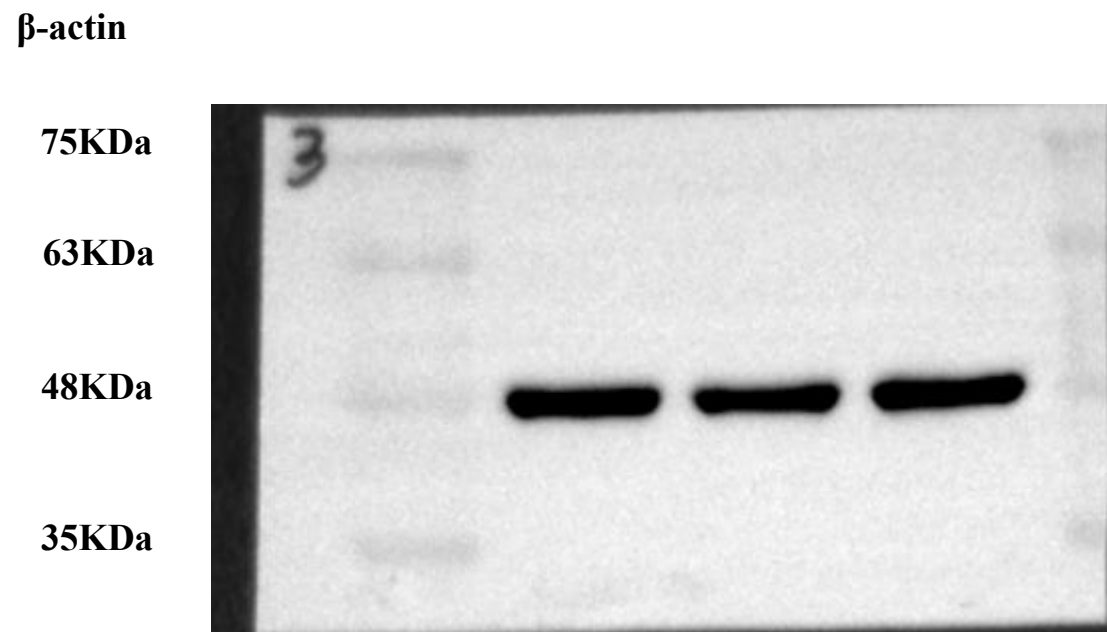

Supplement: Supplementary file 6 — Supplementary material 6 (PDF 101 kb) [file 12033_2023_952_MOESM6_ESM.pdf]
